# X-ray scanning microscopies of microcalcifications in abdominal aortic and popliteal artery aneurysms

**DOI:** 10.1107/S2052252519001544

**Published:** 2019-02-15

**Authors:** C. Giannini, M. Ladisa, V. Lutz-Bueno, A. Terzi, M. Ramella, L. Fusaro, D. Altamura, D. Siliqi, T. Sibillano, A. Diaz, F. Boccafoschi, O. Bunk

**Affiliations:** a Institute of Crystallography, National Research Council, via Amendola 122/O, Bari, Bari 70125, Italy; b Paul Scherrer Institut, Forschungsstrasse 111, Villigen PSI, 5232, Switzerland; c Department of Health Sciences, University of Piemonte Orientale, Via Solaroli 17, Novara, 28100, Italy

**Keywords:** microdiffraction, microcalcifications, tissues, aneurysms, SAXS, WAXS, X-ray scanning microscopy

## Abstract

Abdominal aortic and popliteal artery aneurysms are vascular diseases which show massive degeneration, weakening of the vascular wall and loss of the vascular tissue functionality. They are driven by inflammatory, hemodynamical factors and biological alterations that may lead, in the case of an abdominal aortic aneurysm, to sudden and dangerous ruptures of the arteries. Here, human aortic and popliteal aneurysm tissues were obtained during surgical repair, and studied by synchrotron radiation X-ray scanning microdiffraction and small-angle scattering, to investigate the microcalcifications present in the tissues. Data collected during the experiments were transformed into quantitative microscopy images through the combination of statistical approaches and crystallographic methods. As a result of this multi-step analysis, microcalcifications, which are markers of the pathology, were classified in terms of chemical and structural content. This analysis helped to identify the presence of nanocrystalline hy­droxy­apatite and microcrystalline cholesterol, embedded in myofilament, and elastin-containing tissue with low collagen content in predominantly nanocrystalline areas. The generality of the approach allows it to be transferred to other types of tissue and other pathologies affected by microcalcifications, such as thyroid carcinoma, breast cancer, testicular microli­thia­sis or glioblastoma.

## Abstract 2

Microcalcifications are important pathological markers. Thus, it is relevant to study the occurring crystalline phases, their formation mechanisms, and the correlations between their structure and pathologic modifications of human tissue. Combining scanning X-ray microdiffraction and small-angle scattering reveals the crystalline phases of microcalcifications, and the abundance and orientation of collocated fibrous tissues (elastin, collagen, myofilament), spatially resolved over aneurysm tissue samples.

## 1. Introduction   

An aneurysm is characterized by an abnormal dilatation of blood vessels, caused by a progressive weakening of the vessel wall and disruption of the wall microstructure and/or loss of functionality, which, especially in abdominal aortic aneurysms (AAA), can collapse and cause internal bleeding. Among the most common aneurysms, AAA typically occurs in the infrarenal region of the abdominal aorta, and popliteal artery aneurysm (PAA) takes place in the lower limb, at the popliteal cavity behind the knee.

Several factors are correlated to aneurysms such as aging, hypertension, smoking, hypercholesterolemia and genetic factors. These factors can influence the metabolism of the vascular extracellular matrix (ECM) by altering its structural remodeling (Wagenseil & Mecham, 2009 ▸). The main structural components of ECM in arterial walls are collagen and elastin, present in different amounts depending on the anatomical site and function. In particular, in the aortic adventitia, collagen is organized into discrete fibers providing vessels with tensile strength and flexibility. Clinical evidence suggests a relationship between AAA development and increase of the aortic collagen content and wall stiffness (Alkiviadis *et al.*, 2013 ▸). However, it is not well understood yet whether the impaired collagen catabolism is a triggering factor or just an epi­phenomenon. While in physiological conditions of arterial adventitia, collagen exhibits a three-dimensional wavy structure, it becomes straighter, uncleavable and two-dimensional in the aneurysm wall, probably to accommodate the increase in collagen abundance (Deguchi *et al.*, 2009 ▸). Moreover, aneurysms are characterized by heterogeneous grades of inflammation that are based on infiltrating cells and ECM changes, in particular disruption of elastic fibers, fibrosis and microcalcifications, formed by calcium deposits (Busch *et al.*, 2016 ▸). Calcifications play a key role in the AAA disease (O’Leary, *et al.*, 2015 ▸). Several studies on the mechanical properties of the surrounding fibrous tissues have suggested that microcalcifications may cause strain in the fibrous region and increase the rupture risk of the vessel wall (Buijs *et al.*, 2013 ▸; Inzoli *et al.*, 1993 ▸; Reeps *et al.*, 2013 ▸). Morphological analyses of individual calcium deposits, isolated from AAA tissues, were performed by scanning electron microscopy and by electron-dispersive X-ray spectroscopy, revealing clusters of spherical particles and platelets, rich in calcium phosphate (Marra *et al.*, 2006 ▸).

Microcalcifications are considered early diagnostic markers of several pathologies, especially for different types of cancers where, depending on the calcification composition, they are directly related to the proliferation of cancer cells (Scott *et al.*, 2016 ▸). Therefore, it is relevant to inspect the composition and crystal structure of these calcified deposits by means of quantitative, non-destructive tools.

In the present work, wide-angle X-ray scattering (WAXS) and small-angle X-ray scattering (SAXS) scanning microscopies were performed on human AAA and PAA tissue samples to confirm the presence of microcalcifications, to map their distribution with spatial resolution in the micrometre range, to identify their crystalline structure, and to determine the spatial distribution of elastin, collagen and myofilament.

As a technique, µSAXS/µWAXS scanning microscopy allows mapping of nanoscale and sub-nanoscale structural information spatially resolved over extended areas of square millimetres up to several square centimetres (Bunk *et al.*, 2009 ▸). The sample is raster scanned through a focused X-ray microbeam. While moving at constant speed along a line of this raster scan the detector records a series of two-dimensional microdiffraction data (µSAXS/µWAXS). The resolution at which the reciprocal-space information is recorded as a function of the momentum transfer *q* and the range that is covered, are limited by the size of the X-ray detector. The real-space resolution is limited by the size of the X-ray beam (∼30 µm in the experiment reported here). A statistical analysis of the µSAXS/µWAXS raw data set, realized by means of signal classification (Lutz-Bueno *et al.*, 2018 ▸) allowed us to extract the least-correlated profiles which best represent the data set. The crystallographic interpretation of the selected profiles allowed us to identify the crystal structures in the microcalcifications. In the case of the SAXS data, this analysis provided information on the occurrence of features to be quantified by an analysis of diffraction peaks associated with fibrous tissue.

## 2. Experimental   

### 2.1. Tissues   

Human AAA and PAA tissues were obtained during open-surgical repair from abdominal aorta and popliteal artery, respectively. Informed consent was obtained from all the patients included in the study, according to the local institute’s regulations and policies (ethical committee of the hospital ‘Maggiore della Carità’, Novara, Italy, ref. AVATAR 1.0). After surgery, fresh tissues from the aneurysm wall were fixed and maintained in 10% neutral buffered formalin for further analyses. The sample thickness was ∼1 mm.

### 2.2. µSAXS/µWAXS scanning microscopy   

µSAXS/µWAXS scanning microscopy data were collected at the cSAXS beamline (Bunk *et al.*, 2009 ▸) of the Swiss Light Source at the Paul Scherrer Institut in Villigen, Switzerland. A monochromatic X-ray beam (λ = 0.09116 nm, *E* = 13.6 keV) was focused down to about 26 µm (vertical) and 38 µm (horizontal) full-width at half maximum (FWHM) by a bent mirror and a bent monochromator crystal, respectively. The flat tissue sections, kept hydrated in phosphate-buffered saline within an ultralene (Spex Sampleprep LLC) sachet, were placed perpendicular to the direct beam and raster scanned through the beam spot, while the detector measured the intensity scattered by the currently illuminated sample volume, integrated over the exposure time and across the sample thickness. Measurements were first done in the SAXS geometry and then in the WAXS geometry with step sizes of 40 µm in both horizontal and vertical directions, and exposure times of 0.32 s for SAXS and of 0.25 s for WAXS. To speed up acquisition, data were recorded in a continuous line-scan mode where the sample is moved at constant speed along a vertical line of the two-dimensional raster scan, with a Pilatus 2M pixelated area detector (Henrich *et al.*, 2009 ▸) continuously recording data. µSAXS/µWAXS data were collected at a sample–detector distance of 7089.8/322.85 mm. The range of the momentum transfer *q* covered by the detector over the full azimuthal angle is 0.015–2 nm^−1^ in SAXS and 0.55–36 nm^−1^ in WAXS. The intense (flux ∼ 3 × 10^11^ photons s^−1^) transmitted, *i.e.* unscattered, X-ray beam was blocked from impinging onto the area detector by a beam stop placed close to the center of the detector. For the SAXS experiments, part of the combined X-ray fluorescence and scattering signal of the beam stop was detected using a point detector. This enabled the measuring of the intensity of the transmitted X-ray beam, thus allowing the SAXS data normalization and the generation of X-ray transmission microscopy (XTM) images. Considering the incoming flux and collection times, radiation damage is not expected to occur in the investigated tissues, as it has been qualitatively verified by comparing data recorded in repeated exposures at the same point.

## 3. Data analysis   

### 3.1. Signal-classification method   

**Figure 1:**
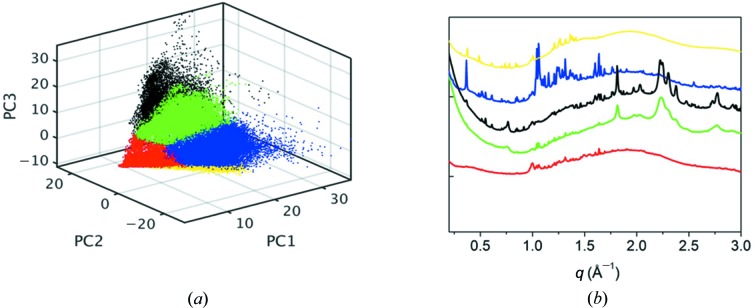
µWAXS signal classification. (*a*) Relative abundance of the principal components (PC1, PC2, PC3) for each profile of the entire µWAXS data set. Clusters of similar properties are highlighted in red, green, black, blue and yellow. (*b*) Representative signals extracted as an average of the furthest points from the centroids in each cluster of (*a*).

Two-dimensional µSAXS/µWAXS raw data were calibrated by using standard powders (silver behenate for SAXS and silicon NIST SRM640b for WAXS) and azimuthally integrated into one-dimensional profiles. The µWAXS and µSAXS data sets were statistically analyzed by means of a signal-classification method which allows finding the least-correlated profiles. In this way, the dimensionality of a multivariate data set is reduced, with minimal loss of information, to a few principal components (here PC1, PC2 and PC3) that can be visualized graphically (Lutz-Bueno *et al.*, 2018 ▸). Fig. 1 ▸(*a*) shows the (PC1, PC2, PC3) scores plot of the entire µWAXS data set, where each data point in the scores plot represents a spectrum with its relative abundance of the principal components. After classification of the µWAXS signals into five clusters, representative signals were extracted as an average of the furthest points from the centroids in each cluster, see Fig. 1 ▸(*b*). These signals were used as input for the canonical correlation analysis (CCA) described in the following section. This approach was repeated for the µSAXS data. In the µSAXS case the representative signals exhibited aspects of mixtures of materials, as for example collagen and elastin, while the signals at individual sample positions exhibited more pronounced features than the results of this analysis. We attribute the higher material selectivity in the case of µWAXS in comparison with µSAXS to two factors: (1) signal classification is not material specific and, in the case of two materials occurring together, it will yield the combination of both signals as distinct information, and (2) filaments like elastin and myofilament exhibit varying spacing in their nanostructure and thus varying SAXS signals. Signal classification can only reveal common aspects of all filament signals rather than the full, unperturbed signal of a specific filament of certain characteristics. Therefore, the representative SAXS signals were only used indirectly to inform the more specific multi modal analysis described below.

### 3.2. Canonical correlation analysis   

**Figure 2:**
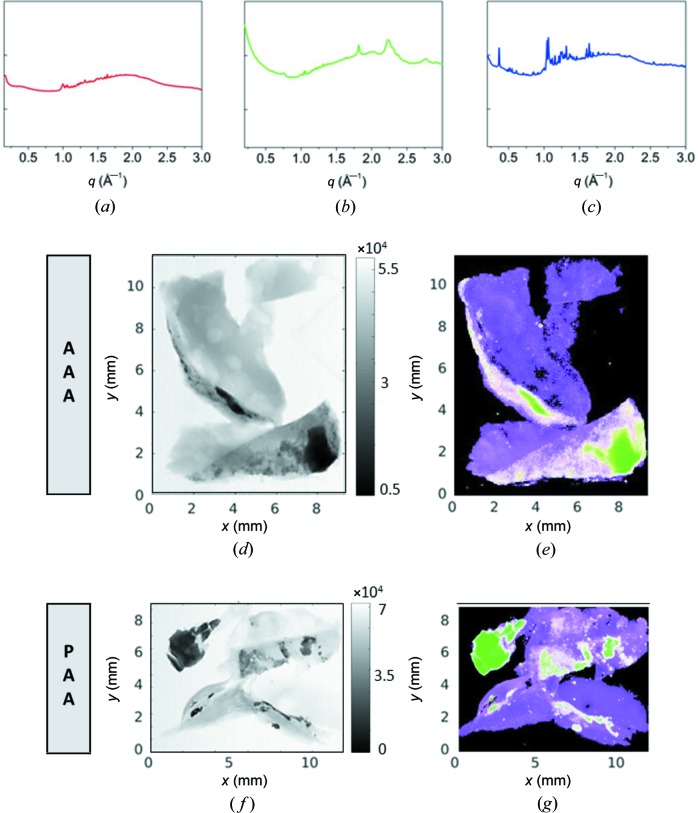
XTM and µWAXS images. Representative µWAXS profiles selected using signal classification followed by canonical-correlation-analysis-based integration: (*a*) amorphous material, (*b*) nanocrystal and (*c*) microcrystal. (*d*), (*f*) XTM images and (*e*), (*g*) µWAXS RGB color maps for the AAA (middle row) and PAA (lower row) tissues. At each point of the RGB color map, the value of the red, green and blue color channel corresponds to the correlation with the representative signal shown in (*a*), (*b*) and (*c*).

The WAXS data selected via signal classification were used as input for the CCA, which is a statistical technique aimed at quantifying the relationship between two sets of variables (Laudadio *et al.*, 2005 ▸). It is a multichannel generalization of ordinary correlation analysis, where the relationship between two arbitrary variables *x* and *y* is quantified by means of the correlation coefficient, a scalar value, ranging between −1 and 1, that measures the degree of linear dependence between *x* and *y*. In ordinary correlation analysis, *x* and *y* are univariate variables. For example, the *x* variable could represent the scattering curve in each pixel of a microscopy image and the *y* variable could represent the model scattering curves (Ladisa *et al.*, 2007 ▸; Guagliardi *et al.*, 2007 ▸). In the present case, the *x* variable represents the µWAXS scanning microscopy data and the *y* variable corresponds to the red (R), green (G), or blue (B) profiles plotted in Figs. 2 ▸(*a*)–2(*c*). These R, G and B profiles were extracted from the least-correlated profiles resulting from the signal classification, see Fig. 1 ▸(*b*), as follows. For each cluster of µWAXS intensity profiles, selected by the signal classification, we summed up the most correlated µWAXS patterns by means of an adapted unsupervised clustering routine (Blatt *et al.*, 1996 ▸, 1997 ▸). This procedure improved the signal-to-noise ratio and completeness of the final µWAXS diffraction patterns, the latter being a fundamental requisite for further analysis of the derived patterns, as explained in Section 4.1. The integrated µWAXS profiles R, G and B in Figs. 2 ▸(*a*)–2(*c*) correspond to the diffraction from an amorphous, a nanocrystalline and a microcrystalline material, respectively. The R curve is a quite broad and unstructured diffraction profile typical for an amorphous material, with a few additional contamination peaks; the G curve is the typical diffraction profile of a nanocrystal, with relatively broad peaks; and the B curve is the diffraction pattern of a microcrystal with sharp, instrumental-resolution-limited, Bragg peaks (Guagliardi *et al.*, 2010 ▸). For visualizing the results of the CCA in Figs. 2 ▸(*e*) and 2(*g*), we utilize a representation of the canonical correlation coefficients as an RGB color map. The relative weight of the coefficients determines the value of the corresponding color channel.

### 3.3. Multi modal imaging   

Each two-dimensional µWAXS and µSAXS data frame was integrated in 16 azimuthal segments, resulting in 16 *I*(*q*) profiles with *I* being the integrated intensity. These data were used to perform a variation of the multi modal analysis described in previous publications (Bunk *et al.*, 2009 ▸; Giannini *et al.*, 2014 ▸). In the multi modal-imaging approach for scanning SAXS data a certain *q* range is selected, corresponding to a certain range of the real-space dimension 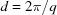. Within this *q* range the average scattering intensity, its variation along the azimuth, and the main orientation of this asymmetric scattering contribution, are determined at each point of the scanning SAXS map. These values are combined in a single plot such that strong isotropic scattering shows up as white, whereas strong asymmetric scattering shows up in a color identifying the azimuthal scattering direction. If, for example, a *q* range is selected that is dominated by scattering from collagen, then a high density of oriented collagen shows up in a bright color with the color corresponding to the orientation, whereas a high density of collagen with isotropic orientation on average over the area illuminated by the X-rays shows up in white. It should be noted that the SAXS signal is caused by the sum of all the materials with a corresponding characteristic feature size. Because of the higher electron-density contrast provided by nano- and microcrystallites in comparison with soft tissue, it can be argued that, in the presence of calcifications or calcified tissue, the SAXS signal of these will dominate. However, even for soft tissue, higher specificity for a material component can be achieved, if characteristic diffraction peaks occur. Examples are the characteristic repeat distance of about 65 nm along the collagen fibers and the higher-order diffraction peaks from this distance, as well as characteristic signals attributed to elastin and myofilament. In such cases the peak position, width and amplitude can be determined in an automated peak-fitting procedure. The peak position is given by the characteristic repeat distance, the width by the distance over which the repeat units are correlated, and the amplitude by the abundance of material and its material-specific scattering power. For the analysis of the µSAXS data in this study, we combined fitting of diffraction peaks with the Fourier analysis previously used for the multi modal analysis (Bunk *et al.*, 2009 ▸). The average, isotropic scattering signal was determined using peak fitting of the azimuthally averaged data. As SAXS intensities exhibit different power laws, depending on the scattering elements being for example, rod-like, platelets or spherical (Glatter & Kratky, 1982 ▸), the automated peak fitting subtracted a background signal 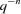 with *n* chosen to have identical intensity on the low- and high-*q* sides of the peak under analysis. At points of the sample where a significant peak signal was detected in the isotropic scattering signal, the amplitude of the asymmetric scattering signal and its main orientation were derived from the simpler Fourier analysis. In this way the multi modal analysis became more robust against differences in intensity of about two orders of magnitude that may occur in the present case, if calcified and not-calcified tissues are compared. Without such precautions, noise in the high-intensity regions may exhibit signal strength comparable to strong peaks in low-intensity regions.

## 4. Results   

In a previous study (Ramella *et al.*, 2018 ▸) it has been shown that healthy aortic and popliteal tissues have a well organized microanatomy. Generally, the classical vascular wall layers (luminal endothelial, medial and adventitia tunicae) are easily distinguishable in histological procedures. In the presence of aneurysms, both tissues show significant thickening of the wall and in particular in aortic aneurysms the collagen content is significantly increased, while elastin is substantially reduced. In popliteal aneurysms and tissues where the microanatomy is significantly altered, collagen and elastin content is quite well preserved. Hematoxylin and eosin staining showed the presence of an inflammatory cell infiltrate predominantly in AAA. Von Kossa staining showed the presence of calcifications in both pathological tissues. The presence of calcifications is typically detected in the late stage of the pathology progression (Ramella *et al.*, 2018 ▸).

### 4.1. µWAXS phase distribution maps   

Figs. 2 ▸(*a*)–2(*c*) show the three representative µWAXS profiles, *i.e.* diffraction patterns, selected following the procedure explained in Sections 3.1 and 3.2. The XTM images are reported in Figs. 2 ▸(*d*) and 2(*f*), along with the result of the CCA [Figs. 2 ▸(*e*) and 2(*g*)] as an RGB color map, for the AAA (middle row) and PAA (lower row) tissues. The XTM images are absorption-contrast maps and provide information on the most absorbing (black) and less absorbing (white) regions in the investigated samples. As calcifications exhibit significantly higher X-ray absorption than soft tissue, regions of the sample with a high density of calcifications will appear as more absorbing for X-rays than regions with a lower density or soft tissue exclusively. Furthermore, if the chemical composition is identical then the higher the density of a calcification, the higher the X-ray absorption. The CCA maps evidence the relative abundance of the amorphous, nano- and microdiffraction patterns. This comparison clearly shows that the darker XTM areas [black in Figs. 2 ▸(*d*) and 2(*f*)], *i.e.* areas of high absorption, contain the nanocrystalline phase [green in Figs. 2 ▸(*e*) and 2(*g*)] as the most abundant component. The dark gray XTM areas, *i.e.* areas of intermediate absorption values, contain mixtures of the amorphous, nanocrystalline and microcrystalline phases, resulting in a white color; and the light gray XTM areas, *i.e.* areas of low absorption, are mixtures of the amorphous and microcrystalline phases, resulting in a magenta color.

**Figure 3:**
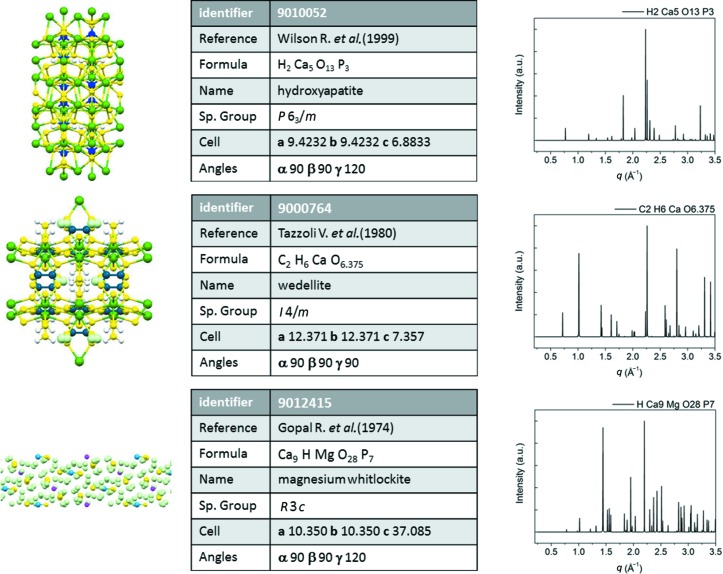
Potential crystal structures of nanocrystalline calcifications. Left column: atomic model; center column: details of the structures from COD database; right column: powder diffraction simulations for an X-ray energy of 13.6 keV.

In order to identify the crystalline structures corresponding to the profiles in Figs. 2 ▸(*b*) and 2(*c*), the Inorganic Crystal Structure Database (ICSD) (Bergerhoff & Brown, 1987 ▸; Belsky *et al.*, 2002 ▸) and the free web Crystallography Open Database (COD) were accessed and, by search/match procedures, two crystal structures were selected which best matched the diffraction patterns: hy­droxy­apatite (ICSD code 187840) for the nano profile in Fig. 2 ▸(*b*) and cholesterol hemi­methano­late (COD code 220-0959) for the micro profile in Fig. 2 ▸(*c*). Among the structures and the corresponding diffraction patterns shown in Fig. 3 ▸, the hy­droxy­apatite structure (COD code 9010052; Wilson *et al.*, 1999 ▸) better reproduces the measured nanocrystalline diffraction pattern, without contributions of weddellite (COD code 9000764; Tazzoli & Domeneghetti, 1980 ▸) and magnesium whitlockite (COD code 9012415; Gopal *et al.*, 1974 ▸). Concerning the microcrystallites, the cholesterol hemi­methano­late (COD code 220-0959) structure was selected among four similar alternatives, listed in Table 1 ▸, by choosing the structure with the highest figure of merit (FOM). Using the selected structures as a model, a whole-profile-fitting approach was adopted to reproduce the experimental diffraction data.

The nanodiffraction pattern in Figs. 2 ▸(*b*) and 4 ▸(*a*) shows the experimental results (red dots) versus the best fit (full black line) and the difference curve (blue curve), as obtained after the Rietveld fitting procedure (Rietveld, 1969 ▸), implemented in the *FullProf* software (Rodríguez-Carvajal, 1993 ▸). The crystalline phase of the investigated sample (ICSD code 187840) was provided to the program, along with the instrumental resolution function determined from the Si NIST standard diffraction data. The inhomogeneous peak broadening of the diffraction peaks was described by a phenomenological model based on a modified Scherrer formula, 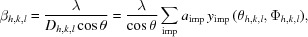
where 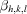 is the crystallite-size contribution to the integral breadth of the (*h*,*k*,*l*) reflection and 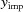 is the real spherical harmonics. After refinement of the 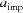 coefficients, the program calculates the apparent size of the crystal domains along each reciprocal lattice vector (*h*,*k*,*l*) direction. Other refinable parameters include the unit-cell parameters. The background was linearly interpolated and subtracted from the experimental data. The refined cell parameters of the hy­droxy­apatite (ICSD code 187840) structure were *a* = *b* = 9.473 Å and *c* = 6.939 Å (α = β = 90°, γ = 120°), the refined crystal domain size was 23 nm and 6 nm along the [002] and [110] crystallographic directions, respectively.

The microcrystalline diffraction pattern in Figs. 2 ▸(*c*) and 4 ▸(*b*) shows the experimental results (red dots) versus the best fit (full black line) and the difference curve (blue curve), as obtained after the Le Bail fitting procedure (Le Bail, 1988 ▸). This procedure is applicable if the experimental peak positions are explained well by a crystal structure, but not their intensities. This fitting procedure has been chosen, as the microcrystalline profile is not complete and the relative peak intensities do not match the proper intensity ratio expected for random powder-like samples, indicative of possible preferred crystalline orientations within the volume illuminated by the X-ray beam. The refined cell parameters of the triclinic cholesterol hemi­methano­late (COD code 220-0959) structure were: *a* = 12.2740 Å, *b* = 34.233 Å and *c* = 6.271 Å (α = 90.223°, β = 93.705°, γ = 91.575°); the crystal domain size was micrometric, *i.e.* larger than the X-ray beam and angular resolution limited.

### 4.2. µSAXS phase distribution maps   

The µSAXS data sets were analyzed using the multi modal approach in combination with peak fitting for the detection of fibrillar material exhibiting distinct diffraction peaks in the SAXS range, as described in Section 3.3.

Aorta and popliteal artery are tissues containing collagen type 1, elastin and myofilament. Collagen type 1 and myo­filament both have a hierarchical organization, with characteristic repeat distances that lead to diffraction peaks within the SAXS range. Collagen type 1 exhibits a series of diffraction peaks corresponding to ∼64–67 nm spacing (Gelse *et al.*, 2003 ▸) and myofilament exhibits a spacing of ∼38–43 nm (Yagi *et al.*, 2004 ▸; Reconditi, 2006 ▸; Reconditi *et al.*, 2014 ▸) in the SAXS range. Elastin fibers are about 100 nm in diameter, with a significant amorphous component (90% core of the fiber) with the remaining 10% made by 5 nm-thick fibrous shell (Ross & Bornstein, 1969 ▸). Consequently, we explored the low-*q* range of the SAXS data set for a corresponding signal. In Figs. 5 ▸(*a*)–5(*c*) a SAXS signal is shown, exhibiting all three signatures, *i.e.* collagen [marked in Fig. 5 ▸(*a*)], a signal at low *q* attributed to elastin [Fig. 5 ▸(*b*)], and myofilament [Fig. 5 ▸(*c*)]. For these plots, the SAXS intensity was multiplied by *q* ^2^ to improve visibility of the peaks. For these peaks we performed multi modal analysis including peak fitting at each point of the samples. The result is shown in Figs. 5 ▸(*d*)–5(*e*) for the AAA and PAA tissues. A hue, saturation, value representation is used. The amplitude of a diffraction peak and thus the abundance of the corresponding fiber is coded as the value *i.e.* brightness, the preferred orientation of the scattering signal related to the orientation of filaments is coded as hue *i.e.* color, and the saturation of the colors relates to the strength of the oriented versus the azimuthally isotropic SAXS signal. The correspondence of color to orientation of the scattering signal is indicated via the color disc.

The entirety of the peak-fitting results for all the investigated points of a sample was used for a simple statistical analysis. The distribution of the positions of the fifth-order collagen peak is plotted in Figs. 6 ▸(*a*) and 6 ▸(*b*). It is centered at 12.91 ± 0.02 nm for AAA and is reproduced by a bimodal distribution with two peaks at 12.87 ± 0.04 nm and 12.90 ± 0.01 nm for PAA. The fifth-order peak has been chosen as optimum between precision and signal strength, as the peak position can be determined with higher accuracy at high *q* than at low *q*, while in the present case the fifth order is exhibiting a stronger and more reliably detectable signal than even higher diffraction orders like the seventh and ninth orders. For elastin the distribution of the fibril diameter is centered at 101 ± 6 nm for AAA [Fig. 6 ▸(*c*)] and 106 ± 11 nm for PAA [Fig. 6 ▸(*d*)]. For the myofilament the distribution of the myofilament axial spacing is centered at 39 ± 1 nm for AAA [Fig. 6 ▸(*e*)] and 41 ± 2 nm for PAA [Fig. 6 ▸(*f*)]. The specified uncertainty range has been determined as FWHM of the peak-position distribution divided by 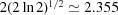, *i.e.* under the assumption of a normal distribution. Differences in lattice spacing for myofilament and elastin in AAA and PAA tissues can be attributed to the relative content and architecture of the connective fibers, which have an overall effect on the elasticity and strength of these tissues, causing several mechanical and functional changes (Tsamis *et al.*, 2013 ▸).

## 5. Discussion   

**Figure 4:**
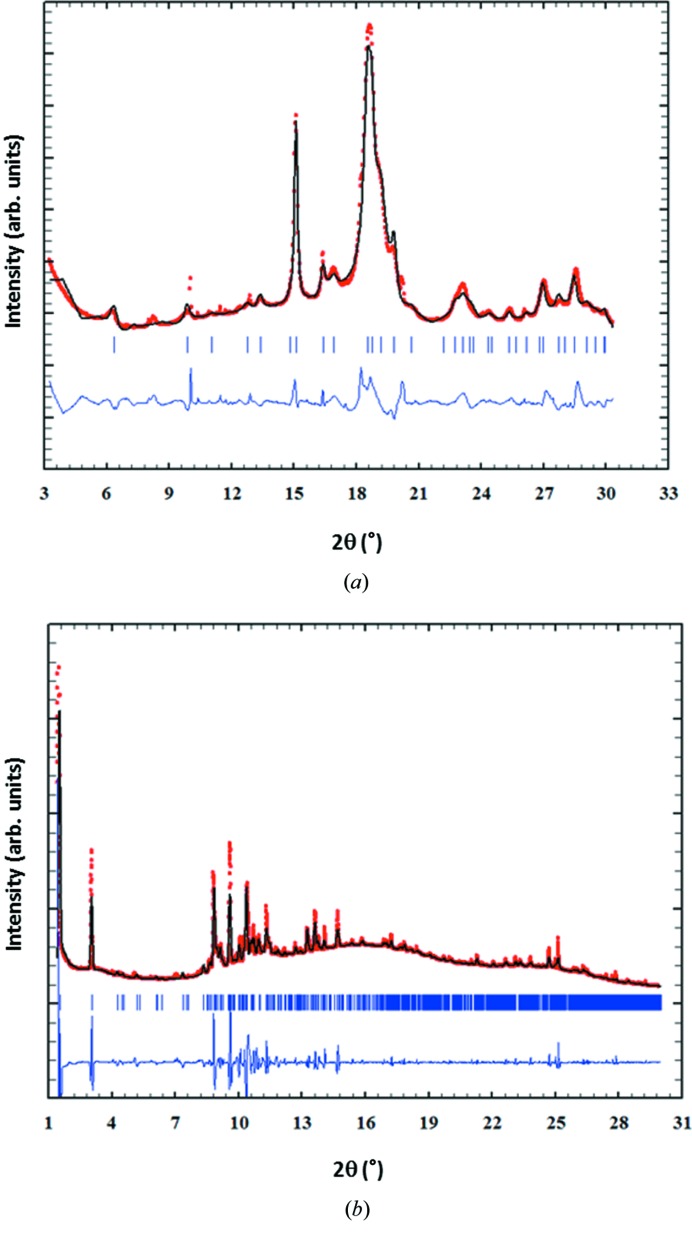
Analysis of nano- and microcrystalline structures. (*a*) Experimental (red dots), best fit (full black line) and the difference curve (blue curve), as obtained after the Rietveld fit of the hy­droxy­apatite structure (ICSD code 187840) and (*b*) experimental (red dots), best fit (full black line) and the difference curve (blue curve), as obtained after the Le Bail fit of the triclinic cholesterol hemi­methano­late (COD code 220-0959).

**Figure 5:**
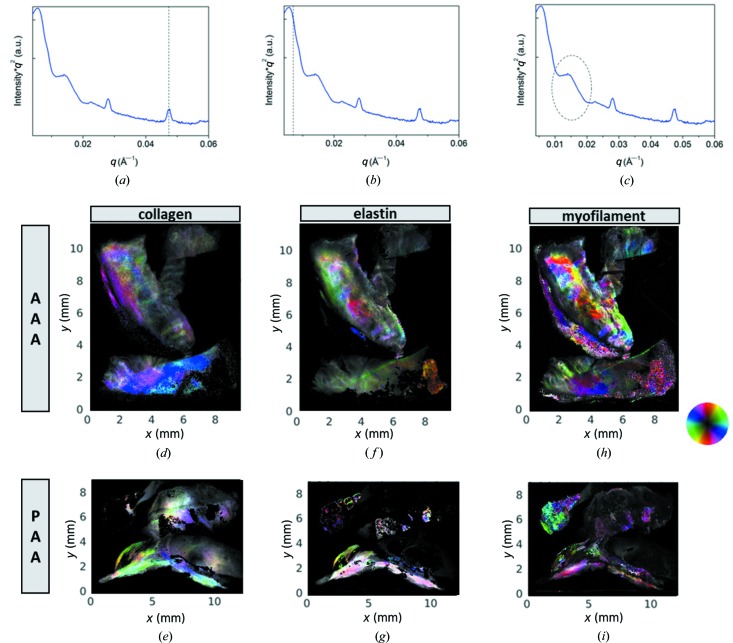
SAXS images. In (*a*), (*b*) and (*c*) identical SAXS profiles are shown, highlighting with markers diffraction peaks of filaments: (*a*) the fifth order of the ∼65 nm axial spacing of collagen type 1, (*b*) a distance attributed to the elastin fibril diameter and (*c*) the first order of the 38–43 nm axial spacing of myofilament. These filament SAXS signals were used in the multi modal analysis of the SAXS patterns; providing images of parameters related to abundance and SAXS orientation of collagen [(*d*) and (*e*)], elastin [(*f*) and (*g*)] and myofilament [(*h*) and (*i*)]. The peak amplitude and thus abundance is coded as brightness. The correspondence of color to orientation of the SAXS signal is indicated via the color disc. Top row abdominal aortic aneurysm (AAA), bottom row popliteal artery aneurysm (PAA).

**Figure 6:**
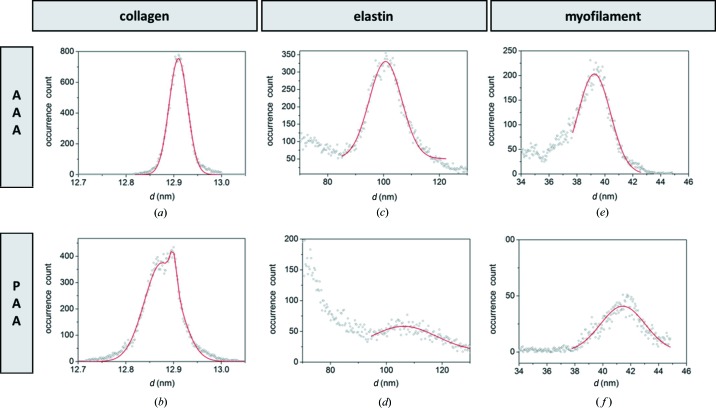
SAXS distribution of structural parameters of filaments. (*a*) Fifth-order collagen peak centered at 12.91 ± 0.02 nm for AAA and (*b*) fitted by two contributions at 12.87 ± 0.04 nm and 12.90 ± 0.01 nm for PAA; (*c*) the elastin-fibril-diameter distribution is centered at 101 ± 6 nm for AAA and (*d*) 106 ± 11 nm for PAA; (*e*) the myofilament lattice spacing is centered at 39 ± 1 nm for AAA and (*f*) 41 ± 2 nm for PAA.

Microcalcifications have been detected in several pathologies, such as vascular diseases (Demer & Tintut, 2008 ▸), thyroid carcinoma (Consorti *et al.*, 2003 ▸; Paschke *et al.*, 2011 ▸), breast cancers (Cox *et al.*, 2012 ▸; Cross *et al.*, 2014 ▸; Naseem *et al.*, 2015 ▸), testicular microli­thia­sis (Renshaw, 1998 ▸) or glioblastoma with oligodendroglial components (Deistung *et al.*, 2013 ▸). In breast tissue, microcalcifications observed have been classified as type I microcalcifications consisting of weddellite crystals, which are of little clinical significance as they are almost exclusively associated to benign lesions, and type II microcalcifications, mainly made of calcium hy­droxy­apatite with intercalated carbonate, closely related with both benign and malignant proliferative lesions (Sathyavathi *et al.*, 2015 ▸). A recent study revealed that, in the case of type II microcalcifications, a relevant role is played by magnesium whitlockite, whose presence increases from benign to invasive cancer (Scott *et al.*, 2016 ▸). In the present study, the nanocrystalline calcifications consist exclusively of hy­droxy­apatite. Concerning the presence of crystalline cholesterol hemi­methano­late [Fig. 4 ▸(*b*)], cholesterol-like signatures were also found in breast tumors colocalized with collagen and apatite aggregates (Kunitake *et al.*, 2018 ▸). Also in our study, comparing the WAXS images shown in Fig. 2 ▸, the cholesterol microcrystals are spatially correlated to hy­droxy­apatite nano-crystals, especially pronounced in the AAA sample, resulting together with the amorphous signal in extended areas of white color. A comparison with the SAXS images in Fig. 5 ▸ indicates that, at least for AAA tissue, collagen fibers coincide with these crystallites. It is striking that the areas with extended nanocalcifications (green in Fig. 2 ▸) seem to be very low in collagen, whereas elastin and myofilament are observed. As these results have been obtained in two-dimensional imaging experiments, different types of tissue could overlay each other in the X-ray beam and thus the corresponding signals would overlap. However, the absence of signals is relevant, as this cannot be explained with a geometrical effect. We thus conclude that nanocalcifications anti-correlate with collagen, but coexist with elastin and myofilament in both the AAA and PAA samples studied.

## 6. Conclusions   

In the overall diagnostic process of several pathologies, microcalcifications are considered a useful indicator of enhanced risk for the patient. In tumors, for example, the crystalline origin of the microcalcifications is fundamental in informing the diagnosis, as it is strongly correlated to the malignancy level of the tumor. X-ray scanning microdiffraction, also called µWAXS, can reveal the presence of crystalline, nanocrystalline and amorphous phases in micrometric areas, and map them across extended areas. In this work, we employed this technique on aortic and popliteal aneurysms to identify the chemical and structural nature of their microcalcifications, to map their distributions across the tissues, and via µSAXS mapping, to reveal the concomitant abundance and orientation of soft-tissue-fiber constituents like collagen, elastin and myofilament. Our findings include that the observed nanocalcifications consist of hy­droxy­apatite and the microcalcifications consist of cholesterol. Furthermore, nanocalcifications appear to be anti-correlated with collagen, whereas myofilament and nanocalcifications have been observed concurrently.

Although AAA and PAA have similar symptoms in terms of vessel-wall weakening, related to the loss of the biomechanical properties of the medial layer, the pathogenesis and progression of aneurysms located in different anatomical regions still need further study. Differences have already been demonstrated (Hurks *et al.*, 2014 ▸; Ramella *et al.*, 2018 ▸), such as the significant presence of an inflammatory infiltrate and the severe ECM degradation in AAAs, in comparison to PAAs.

The present work shows a new approach, able to reveal structural differences on the nanometre and sub-nanometre scale with microscopic spatial resolution, potentially helping to better distinguish between different pathological progressions and contributing to the understanding of them. The same approach can be extended to other pathologies or similar health problems, as specific sample preparation like labeling, or dedicated sample environments are not required.

## Figures and Tables

**Table 1:** Cholesterol structures screened to explain the experimental micro­diffraction profile in Fig. 2 ▸(*b*) During screening the structure with the highest FOM was chosen.

Number	COD code	Structure name	Chemical formula	FOM
1	00-220-0959	2:1 Cholesterol–hemi­methano­late	C_27_H_46_O·0.5CH_4_O	0.57
2	00-450-2852	2:1:1 Cholesterol–benzyl alcohol–water	2C_27_H_48_O·C_7_H_8_O·H_2_O	0.47
3	00-720-2351	2:1 Cholesterol–4-iodo­phenol cocrystal	2C_27_H_46_O·C_6_H_5_IO	0.46
4	00-450-2848	2:1 Cholesterol–butanol	2C_27_H_46_O·C_4_H_10_O	0.44
